# Exploring the Impact of First Trimester Elevated Lipoprotein(a) Levels on Preeclampsia, Preterm Delivery, and Fetal Growth Restriction

**DOI:** 10.3390/jcm14124134

**Published:** 2025-06-11

**Authors:** Apostolia Galani, Athanasios Zikopoulos, Anastasios Potiris, Efthalia Moustakli, Stefania Maneta-Stavrakaki, Maria Paraskevaidi, Charikleia Skentou, Konstantinos Zikopoulos, Peter Drakakis, Sofoklis Stavros

**Affiliations:** 1 Department of Metabolism, Digestion and Reproduction, Faculty of Medicine, Imperial College London, London W12 ONN, UK; l.galani22@imperial.ac.uk (A.G.); m.paraskevaidi@imperial.ac.uk (M.P.); 2 Third Department of Obstetrics and Gynecology, University General Hospital “ATTIKON”, Medical School, National and Kapodistrian University of Athens, 12462 Athens, Greece; thanzik92@gmail.com (A.Z.); apotiris@med.uoa.gr (A.P.); pdrakakis@med.uoa.gr (P.D.); 3 Laboratory of Medical Genetics, Faculty of Medicine, School of Health Sciences, University of Ioannina, 45110 Ioannina, Greece; ef.moustakli@uoi.gr; 4 Imperial College Healthcare NHS Trust, London W12 1NY, UK; s.maneta-stavrakaki17@imperial.ac.uk; 5 Department of Obstetrics and Gynecology, Medical School, University of Ioannina, 45110 Ioannina, Greece; haraskentou@uoi.gr (C.S.); kzikop22@gmail.com (K.Z.)

**Keywords:** first trimester, gestational hypertensive disorders, pre-eclampsia, pregnancy, lipoprotein(a)

## Abstract

**Background/Objectives:** Preeclampsia (PE), characterized by its complex and multisystemic nature, significantly compromises the health outcomes of both mothers and their newborns. According to recent research, its underlying pathophysiological mechanisms may be influenced by abnormalities in lipid metabolism. The purpose of this study is to assess the association between unfavorable pregnancy outcomes and increased lipoprotein(a) levels in the first trimester and the subsequent risk of PE. **Methods:** A prospective cohort study comprising 150 pregnant women with a gestational age of less than 12 weeks and no history of PE was carried out at the University Hospital of Ioannina. In the first trimester, lipoprotein(a) levels were assessed, and individuals were monitored for the emergence of preeclampsia, preterm birth, gestational hypertension without proteinuria, and fetal growth limitation. Selection bias was minimized through the use of sequential sampling and rigorous inclusion and exclusion criteria. Associations were assessed using logistic regression analysis. **Results:** Women with elevated lipoprotein(a) levels had a considerably greater risk of PE than those with normal levels (64.7% vs. 15.5%, *p* < 0.001). Additionally, elevated lipoprotein(a) was linked to higher odds of fetal growth restriction (*p* < 0.001), gestational hypertension without proteinuria (*p* = 0.024), and premature delivery (*p* = 0.009). These results imply that early lipoprotein(a) screening during pregnancy may help identify women who are at high risk for PE and its associated negative consequences. **Conclusions:** The association between PE and elevated first-trimester lipoprotein(a) levels highlights the necessity for a deeper understanding of the underlying pathophysiological mechanisms, which could ultimately improve outcomes for both mothers and newborns.

## 1. Introduction

Preeclampsia (PE), a complicated, multisystem illness, is a major global cause of morbidity and mortality and a threat to the health of both mothers and newborns. Clinically, PE is characterized by the onset of hypertension, typically defined as a blood pressure exceeding 140/90 mmHg, occurring after 20 weeks of gestation and often accompanied by proteinuria. PE, which affects 2% to 8% of pregnancies worldwide, is a significant challenge to obstetric care because of its unpredictable course and link to serious consequences such as eclampsia, multi-organ failure, and elevated maternal and fetal mortality [1,2].

The pathophysiology of PE is multifaceted and remains incompletely understood, although current evidence indicates that environmental influences, dysregulated immune responses, and genetic susceptibility contribute interactively to its onset. Widespread endothelial dysfunction, resulting in diminished placental perfusion and systemic vascular abnormalities, is a key factor in the pathogenesis of PE [3,4]. Recent research has underscored the importance of metabolic factors, particularly lipid metabolism abnormalities, in the pathogenesis of PE. Dyslipidemia and increased oxidative stress (OS), key alterations in lipid metabolism during pregnancy, have been associated with the development of inflammation and vascular damage in affected women [5,6,7].

Increasingly acknowledged as a significant lipid component, lipoprotein(a) is a unique lipoprotein particle that structurally resembles low-density lipoprotein (LDL) but differs in that it contains apolipoprotein(a). Atherosclerosis and thrombosis are two cardiovascular diseases for which elevated lipoprotein(a) levels are known independent risk factors. They possess pro-inflammatory and pro-atherogenic properties [8,9]. Examining biomarkers that affect inflammation and endothelial function, including lipoprotein(a), may shed light on the vascular pathology that underlies spontaneous preterm birth and fetal growth restriction (FGR) [10,11].

Preterm delivery, FGR, and other negative perinatal consequences are among the major concerns that PE poses to the health of the mother. Both conditions significantly impact neonatal health, contributing to increased morbidity and mortality, as well as long-term effects including elevated risks of respiratory distress syndrome, neurodevelopmental disorders, and metabolic syndrome in adulthood [12,13]. Since vascular pathology is a key factor in FGR and spontaneous preterm birth, studying biomarkers like lipoprotein(a) that affect endothelial function and inflammation could provide valuable insights into the underlying mechanisms and support early diagnosis [10,11].

Although there is growing evidence that lipoprotein(a) is associated with cardiovascular risk and vascular dysfunction, its potential as a predictive biomarker for PE during pregnancy has not been properly investigated. This gap hinders the potential for early detection and timely intervention in high-risk pregnancies. Accordingly, this study aims to assess the relationship between increased lipoprotein(a) levels in the first trimester and the subsequent risk of PE, spontaneous preterm delivery, and fetal growth restriction. Improving our understanding of this relationship may facilitate the development of more effective screening techniques and targeted treatments, ultimately improving outcomes for both mothers and newborns.

## 2. Materials and Methods

### 2.1. Study Design

This prospective cohort study was conducted at the University Hospital of Ioannina from January 2021 to December 2022. A total of 150 pregnant women presenting for their first-trimester prenatal visit with a gestational age less than 12 weeks and no history of pre-eclampsia were enrolled in the study. All participants provided a written informed consent for participation and publication of the results. The study was conducted in accordance with the Declaration of Helsinki and approved by the Ethics Committee of University Hospital of Ioannina with protocol identifier 27924 and approval date 14 October 2019.

**Figure 1:**
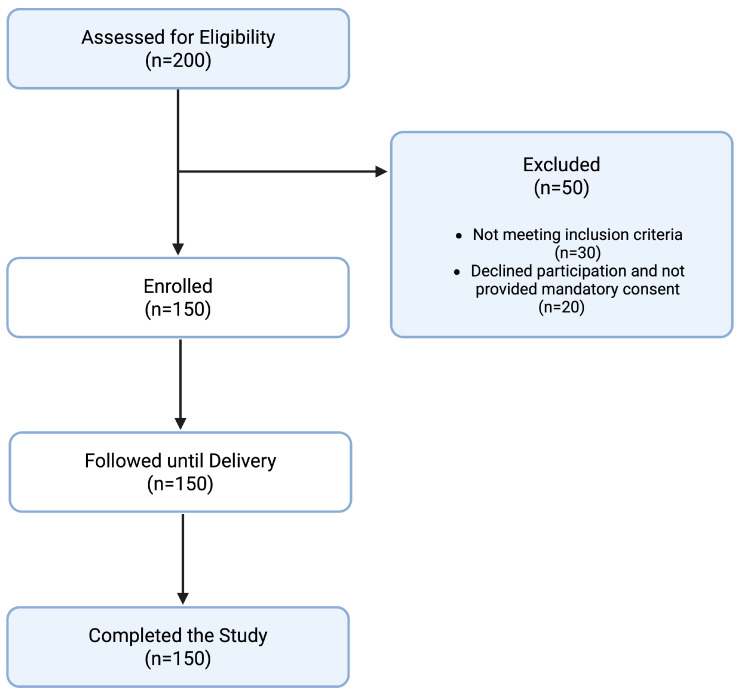
Flowchart of participant inclusion.

A flowchart detailing the study design and participant inclusion is presented in Figure 1. The baseline characteristics of the study sample are presented in Table 1. Selection bias was minimized by consecutive sampling and adherence to strict inclusion/exclusion criteria.

### 2.2. Eligibility Criteria

Eligible participants were women with a singleton pregnancy and a confirmed gestational age of less than 12 weeks at the time of enrollment. Women aged 18 to 45 years were included; the mean maternal age was 29.5 ± 4.3 years. Both nulliparous and multiparous women were enrolled, with 45% being nulliparous. Participants with chronic diseases—including pre-existing hypertension, diabetes mellitus, renal disease, autoimmune disorders, or thyroid dysfunction—were excluded. Women with a history of pre-eclampsia or multiple gestations were also excluded to minimize potential confounding. Racial or ethnic classification was not used as an inclusion criterion or for stratification, though self-reported demographic data were collected. All participants were enrolled before 12 weeks of gestation.

### 2.3. Measurements and Outcomes

Blood samples for measuring lipoprotein(a) levels were collected using a standardized kit (lipoprotein(a) Quantification Kit, XYZ Biotech, Randolph, NJ, USA) as per manufacturer instructions. Elevated lipoprotein(a) was defined as levels greater than the 90th percentile, aligning with thresholds used in similar studies cited in the current literature. Uterine artery Doppler ultrasound was performed to assess placental blood flow. The primary outcome was the development of PE, defined according to the International Society for the Study of Hypertension in Pregnancy (ISSHP) criteria [14]. Secondary outcomes included hypertension without proteinuria, preterm delivery (before 37 weeks of gestation), and fetal growth restriction. All participants completed the follow-up period and were included in the final analysis.

### 2.4. Statistical Analysis

Statistical analyses were performed using SPSS version 26.0. The association between elevated lipoprotein(a) levels and pregnancy outcomes was assessed using logistic regression models, providing odds ratios (ORs) and 95% confidence intervals (CIs). *p*-values less than 0.05 were considered statistically significant. Each outcome was adjusted for potential confounders, such as age, BMI, smoking status, and parity. Interaction and subgroup analyses were conducted to explore the robustness of the findings.

### 2.5. Sample Size and Power Calculation

A priori sample size estimation was conducted based on preliminary data suggesting a 15% baseline incidence of preeclampsia and a projected 60% incidence among individuals with elevated lipoprotein(a). Using a power of 80% and alpha of 0.05, a minimum of 28 individuals in the high lipoprotein(a) group was needed to detect a statistically significant difference. The final cohort size exceeded this threshold, providing confidence in the study’s internal validity.

### 2.6. Data Management and Quality Control

All data were anonymized and stored in a secure, password-protected electronic database compliant with GDPR standards. Data integrity was ensured through double-entry verification and periodic audits by an independent reviewer. Quality control procedures included inter-assay and intra-assay variability checks for lipoprotein(a) quantification and cross-validation with a secondary biochemical platform for a random 10% sample subset.

## 3. Results

Our research revealed a strong correlation between high levels of lipoprotein(a) in the first trimester and unfavorable pregnancy outcomes, including the emergence of PE. Among the 150 participants, 34 women (22.7%) exhibited elevated lipoprotein(a) levels (≥30 mg/dL). The prevalence of PE was significantly higher in this subgroup (64.7%) compared to those with normal lipoprotein(a) levels (15.5%) (Table 2). After adjusting for maternal age, body mass index (BMI), and parity, logistic regression analysis yielded an odds ratio (OR) of 9.47 (95% CI: 3.98–22.54; *p* < 0.001), indicating a strong and independent association between elevated lipoprotein(a) levels and the risk of PE.

Our research revealed a strong correlation between high levels of lipoprotein(a) in the first trimester and unfavorable pregnancy outcomes, including the emergence of PE. Fetal growth restriction (FGR) was defined as a birth weight below the 10th percentile for gestational age, confirmed by neonatal measurements after delivery. Preterm birth was classified as delivery before 37 weeks of gestation and further categorized into spontaneous preterm labor or medically indicated delivery based on clinical records. Preeclampsia cases were classified as early-onset if diagnosed before 34 weeks and late-onset if diagnosed at or after 34 weeks of gestation. The gestational age at PE diagnosis was recorded for all affected participants. Among the 150 participants, 34 women (22.7%) exhibited elevated lipoprotein(a) levels (≥30 mg/dL).

High levels of lipoprotein(a) were significantly associated with pre-eclampsia and other adverse pregnancy outcomes. A total of 17.6% of women in the elevated lipoprotein(a) group had hypertension without proteinuria, compared to 5.2% in the normal group (adjusted OR: 3.90; 95% CI: 1.20–12.63; *p* = 0.024). Women exhibiting elevated lipoprotein(a) levels demonstrated a significantly increased risk of preterm delivery compared to those with normal levels (10.3%), reflected by an adjusted odds ratio of 4.77 (95% CI: 1.88–12.09; *p* = 0.009). Furthermore, the prevalence of FGR was significantly higher in the cohort with elevated lipoprotein(a) levels (55.9%) relative to those with normal levels (14.7%), with an adjusted odds ratio of 7.27 (95% CI: 3.02–17.49; *p* < 0.001).

Analysis of lipoprotein(a) levels within the study population showed that almost 23% of participants had values above the clinically relevant cutoff of 30 mg/dL (Table 3). This finding underscores the potential value of using this cutoff to identify pregnancies at higher risk for adverse outcomes.

These results reinforce and expand upon prior studies associating vascular dysfunction and lipid disturbances with negative pregnancy outcomes. Although earlier studies have reported associations between dyslipidemia and PE [15,16,17], few have specifically evaluated lipoprotein(a) levels in early pregnancy as a predictive biomarker. In light of our findings, elevated lipoprotein(a) levels in high-risk pregnancies may serve as a valuable early marker for informing more intensive prenatal care and guiding potential therapeutic strategies. According to lipoprotein(a) status, Table 4 shows the relative risks of adverse outcomes. The significance and consistency of the relationships emphasizes how crucial it is to include lipoprotein(a) in prenatal risk assessment. Additional studies are needed to evaluate the clinical utility and economic feasibility of routine lipoprotein(a) screening during pregnancy, and to confirm these findings in larger and more ethnically diverse cohorts.

## 4. Discussion

Meta-analytic evidence indicates that elevated lipoprotein(a) levels may aggravate endothelial dysfunction by interacting synergistically with cardiovascular risk factors, including homocysteine and high-sensitivity C-reactive protein (hsCRP) [12,13,18]. These pathways may exert synergistic effects during pregnancy, particularly in individuals with metabolic comorbidities or underlying genetic predispositions.

Given the known interactions between elevated lipoprotein(a) levels and traditional cardiovascular risk factors, it is plausible that lipoprotein(a) could serve as a particularly valuable predictive biomarker for pre-eclampsia in women with preexisting conditions such as hypercholesterolemia, diabetes, or cardiovascular disease. These conditions already predispose individuals to endothelial dysfunction and heightened inflammatory states, which may be further aggravated by elevated lipoprotein(a), increasing the likelihood of developing PE. Therefore, incorporating lipoprotein(a) assessment into first-trimester screening may be especially beneficial in these high-risk populations.

Additionally, the pro-inflammatory and atherogenic attributes of lipoprotein(a) have the potential to impact the fundamental processes of healthy placental development, specifically trophoblast invasion and spiral artery remodeling [11]. Disturbances in these pathways have been consistently implicated in early-onset PE, which is commonly associated with heightened risks of adverse maternal and fetal outcomes [10,17]. Immune tolerance may be disrupted, and systemic vascular resistance promoted by elevated lipoprotein(a) levels, which may also influence molecular pathways at the maternal-fetal interface.

Another critical factor is the potential role of lipoprotein(a) in modulating OS within the placenta [15,16]. Increased OS in the placenta has been linked to the release of anti-angiogenic proteins such as endoglin and soluble fms-like tyrosine kinase-1 (sFlt-1), which are known to disrupt normal endothelial function and cause proteinuria and hypertension [19,20,21]. More research is necessary to fully understand how lipoprotein(a) interacts with these angiogenic regulators.

Our study reinforces the role of lipoprotein(a) as a key contributor to the pathophysiology of PE, demonstrating a strong association between elevated levels in early pregnancy and the subsequent development of the condition. PE may share similar underlying pathogenic mechanisms, as these findings support earlier research that identified increased lipoprotein(a) as a risk factor for cardiovascular illnesses [22,23].

Elevated lipoprotein(a), owing to its pro-inflammatory and pro-oxidative characteristics, has been implicated in the development of atherogenesis, emphasizing its contribution to the endothelial dysfunction associated with PE [24,25]. A defining pathogenic feature of PE and cardiovascular diseases is endothelial dysfunction, which manifests through elevated OS, increased inflammatory signaling, and impaired vasodilatory response [26,27]. Therapeutic interventions aimed at reducing lipoprotein(a) levels may prove beneficial for patients at risk of PE, given the shared pathophysiological mechanisms involved [28,29]. With a 64.7% incidence of PE in the high lipoprotein(a) group, our study’s substantial correlation between raised lipoprotein(a) levels and PE is very remarkable. This incidence rate indicates that lipoprotein(a) could function as a significant prognostic marker for PE, due to its markedly greater frequency relative to that observed in the general pregnant population. Women with increased lipoprotein(a) levels are more likely to experience preterm delivery, fetal growth restriction, and hypertension without proteinuria, contributing to the complicated nature of PE as a systemic condition.

There are probably a variety of different ways whereby high lipoprotein(a) levels lead to PE. It is known that lipoprotein(a) inhibits fibrinolysis and results in a prothrombotic state that exacerbates inflammation and endothelial damage by blocking the conversion of plasminogen to plasmin [30,31]. Additionally, apolipoprotein(a) and lipoprotein (a) may bind to extracellular matrix components more easily due to their unique structures, which could accelerate vascular damage and increase OS [32,33].

Our study also highlights the need for further research into the genetic factors underlying elevated lipoprotein(a) levels and their interaction with other risk factors for pre-eclampsia. Given the strong genetic determination of lipoprotein(a) levels by variants in the LPA gene, identifying these genetic factors may help predict susceptibility to pre-eclampsia and support the development of individualized preventive and therapeutic interventions [34,35]. Certain polymorphisms in the LPA gene have been linked by genetic research to higher levels of lipoprotein(a) and a higher risk of cardiovascular disease; these polymorphisms may also raise the risk of pre-eclampsia [36,37].

Although our findings provide new insights, several limitations should be acknowledged. Although the sample size was adequate to detect statistically significant differences, it may not fully represent the diversity of ethnic populations [38]. Additionally, the lack of serial lipoprotein(a) measurements during pregnancy restricts our ability to assess dynamic changes in lipid metabolism throughout gestation. The evidence base would be more robust if future research included longitudinal sampling and investigated genetic variants associated with lipoprotein(a) levels.

The addition of lipoprotein(a) to multifactorial risk prediction models may increase prognosis accuracy, given the multifactorial etiology of PE. In addition to established markers like pregnancy-associated plasma protein A (PAPP-A) and placental growth factor (PlGF), incorporating lipoprotein(a) measurement into first-trimester screening may enhance the accuracy of early risk assessment and support more personalized prenatal care.

Our findings have important therapeutic implications, suggesting that measuring lipoprotein(a) levels in the first trimester could enhance early identification of women at high risk for PE. This strategy may facilitate rigorous surveillance and the timely deployment of therapeutic interventions to attenuate the incidence of adverse outcomes. Particularly, extensive multicenter research is required to corroborate our findings and to evaluate the cost-effectiveness of incorporating lipoprotein(a) screening into prenatal care, thereby further defining its value as an independent prognostic biomarker for PE. Lipoprotein(a) measurement may be useful in predicting PE, but it may also offer important insights into managing other pregnancy-related issues. Considering the association between elevated lipoprotein(a) levels and adverse outcomes such as fetal growth restriction and preterm delivery, screening for lipoprotein(a) may aid in identifying at-risk pregnancies and support the implementation of more individualized and proactive care strategies.

## 5. Limitations of the Study

While this study contributes valuable knowledge on the association between elevated lipoprotein(a) levels during the first trimester and negative pregnancy outcomes, it is important to recognize several limitations. Although our analyses adjusted for key confounding variables such as maternal age, body mass index, parity, and smoking status, residual confounding from unmeasured factors—including pre-existing comorbidities and socioeconomic variables—cannot be entirely excluded. The results may not be as applicable to larger groups with different demographic, genetic, and environmental backgrounds because the study was limited to a single tertiary care facility. Although sequential sampling mitigated selection bias, the sample may not be fully representative of all pregnant individuals, particularly those receiving care in non-hospital or rural settings. The external validity of our findings may be influenced by variations in healthcare access, socioeconomic status, and ethnicity, all of which could affect lipoprotein(a) levels and pregnancy outcomes.

Furthermore, although the study was well powered to detect variations in pre-eclampsia rates, the sample size remains relatively small. This limitation restricts the possibility of performing in-depth subgroup analyses, including stratification by pre-eclampsia severity, onset timing, or associated comorbidities. Rare yet clinically significant events, such as stillbirth or early-onset fetal growth restriction, may not have been sufficiently captured. Further investigation into these relationships and validation of our findings require larger, multi-center research with varied populations.

Additionally, residual confounding from unmeasured factors cannot be entirely excluded, despite accounting for several confounders such as age, BMI, smoking status, and parity. Potential determinants of lipoprotein(a) concentrations and pregnancy-related risks, including socioeconomic status, dietary patterns, stress, and genetic factors, were not accounted for in this study. Moreover, lipoprotein(a) levels were assessed only once in the first trimester, potentially overlooking the dynamic fluctuations that arise throughout pregnancy as a result of physiological changes. Serial assessments may provide a more comprehensive understanding of lipoprotein(a)’s role in pregnancy progression and risk stratification. Furthermore, reproducibility and comparability between studies may be impacted by variations in laboratory procedures and test sensitivity, even when the assay utilized was validated and standardized.

Ultimately, without examining the influence of clinical treatments based on lipoprotein(a) levels, this study was limited to investigating the relationship between lipoprotein(a) and adverse outcomes. Therefore, while elevated lipoprotein(a) may serve as a potential predictive biomarker, its effectiveness in informing clinical decision-making for preventive therapies or enhanced monitoring has not been conclusively demonstrated. Future studies should examine whether monitoring of lipoprotein(a) during pregnancy enhances patient acceptance, cost-effectiveness, and mother and fetal outcomes.

## 6. Conclusions

In conclusion, our study adds to the expanding evidence regarding the role of lipid metabolism in pregnancy and its critical influence on maternal and fetal health by demonstrating a significant association between elevated first-trimester lipoprotein(a) levels and the development of PE. We identified a significant association between elevated first-trimester lipoprotein(a) levels and the onset of pre-eclampsia, alongside increased risks of preterm delivery and fetal growth restriction. These findings suggest that lipoprotein(a) could serve as an early biomarker for identifying pregnancies at increased risk of diverse adverse outcomes.

Pregnancy problems and cardiovascular disease share vascular and inflammatory pathways, underscoring the urgent need to comprehend the underlying pathophysiological mechanisms. More extensive, multi-center investigations are necessary to confirm these results and evaluate the practicality, affordability, and clinical usefulness of integrating lipoprotein(a) screening into standard prenatal care. In conclusion, the outcomes for mothers and infants can be enhanced through the early detection and focused management of high-risk pregnancies.

## Figures and Tables

**Table 1:** Baseline characteristics of the study population.

Characteristic	Mean ± SD/%
Age (years)	29.5 ± 4.3
BMI (kg/m^2^)	24.8 ± 3.5
Smoking (%)	22%
Parity (nulliparous %)	45%

BMI: Body Mass Index.

**Table 2:** Comparison of adverse pregnancy outcomes between women with elevated and normal first-trimester lipoprotein(a) levels. Data are presented as number of cases with percentages in parentheses. Elevated lipoprotein(a) was defined as ≥30 mg/dL. Statistical significance was assessed using chi-square tests.

Outcome	Elevated Lp(a) (%) (n = 34)	Normal Lp(a) (%) (n = 116)	*p*-Value
Pre-eclampsia	22 (64.7%)	18 (15.5%)	<0.001 *
Hypertension without proteinuria	6 (17.6%)	6 (5.2%)	0.024 **
Preterm delivery	12 (35.3%)	10 (10.3%)	0.009 ***
Fetal growth restriction	19 (55.9%)	17 (14.7%)	<0.001 *

Lp(a): Lipoprotein (a), * indicates statistical significance at the level of 0.1%, ** indicates statistical significance ath the level of 5% and *** indicates statistical significance at the level of 1%.

**Table 3:** Distribution of participants according to lipoprotein(a) levels categorized by concentration ranges (mg/dL). Values represent the number of participants and their corresponding percentages within each category.

Lp(a) Range (mg/dL)	Number of Participants	Percentage
<30	116	77.3%
≥30	34	22.7%

Lp(a): Lipoprotein (a).

**Table 4:** Comparison of pregnancy outcomes between elevated and normal lipoprotein(a) levels. Odds ratios (OR) with 95% confidence intervals (CI) for the association between selected pregnancy outcomes and the studied exposure.

Outcome	OR (95% CI)	*p*-Value
Pre-eclampsia	9.47 (3.98–22.54)	<0.001 *
Hypertension	3.90 (1.20–12.63)	0.024 **
Preterm Delivery	4.77 (1.88–12.09)	0.009 ***
Fetal Growth Restriction	7.27 (3.02–17.49)	<0.001 *

OR: Odds Ratio, * indicates statistical significance at the level of 0.1%, ** indicates statistical significance ath the level of 5% and *** indicates statistical significance at the level of 1%.

## Data Availability

The raw data supporting the conclusions of this article will be made available by the corresponding author on request.
